# International Society for Pharmacoepidemiology in Action: Advancing Public Health Through Evidence‐Based Advocacy

**DOI:** 10.1002/pds.70466

**Published:** 2026-09-11

**Authors:** G. Sarri, J. Diaz‐Decaro, M. Burcu, M. Soriano Gabarró, R. Gini, P. Bahri, M. E. Ritchey

**Affiliations:** ^1^ Innovative Statistics, Evidence, Value, Access and Health Policy Cytel Inc. London UK; ^2^ Black Swan Causal Labs, LLC Los Angeles California USA; ^3^ Epidemiology Merck & Co. Inc. Rahway New Jersey USA; ^4^ Independent Researcher Potsdam Germany; ^5^ Pharmacoepidemiology Unit ARS Toscana Firenze Italy; ^6^ Pharmacovigilance European Medicines Agency (EMA) Amsterdam the Netherlands; ^7^ Med Tech Epi LLC Philadelphia Pennsylvania USA; ^8^ Center for Pharmacoepidemiology and Treatment Science, Institute for Health, Health Care Policy, and Aging Research & Center for Health Outcomes, Policy, and Economics, Rutgers University New Brunswick New Jersey USA

**Keywords:** advocacy, evidence policy‐making, ISPE, pharmacoepidemiology, public health

## Abstract

- Public health faces increasingly complex challenges for pharmacoepidemiology: monitoring and evaluating the safety and effectiveness of medicines in real‐world populations, predicting and responding to emerging diseases and outbreaks, overcoming inequities in drug access and outcomes, managing risks from widespread medication use, and countering misinformation about treatments. These challenges are further intensified by financial constraints and shifting geopolitical pressures.
- Scientific and professional organizations such as the International Society for Pharmacoepidemiology (ISPE) play a critical advocacy role. ISPE advocates for the effective translation of scientific evidence into policy and healthcare practice. This is driven by purposeful, sustained efforts that bridge evidence and action across health systems and stakeholders around the globe.

## Plain Language Summary

Public health is dealing with many challenges today, such as new diseases, climate change, aging populations, growing health inequalities, and misinformation about the benefits and risks of medicines and devices. To protect people's health, scientific evidence needs to drive public policies and practice, and this requires active and ongoing advocacy. ISPE plays an important role in this work by helping ensure that research on how medicines are used and how effective and safe they are can guide decisions made by regulators, clinicians, public health and other health policy makers. Through a transparent, member‐driven process and strong partnerships, ISPE leads advocacy efforts worldwide and continues to build capacity so that scientific evidence can better support public health.

## 1 Introduction

Public health faces increasingly complex challenges with direct implications for pharmacoepidemiology, including monitoring and evaluating the safety and effectiveness of medicines in real‐world populations, predicting, characterizing and responding to emerging diseases and outbreaks, overcoming inequities in drug access and outcomes, managing risks associated with widespread medication use, and countering misinformation about treatments [1, 2, 3, 4]. These challenges are further intensified by financial constraints and shifting geopolitical dynamics. Together, these pressures highlight the growing need for coordinated, evidence‐driven responses that can translate pharmacoepidemiologic insights into effective and equitable public health policies and practice [5]. That translation does not happen automatically; it requires deliberate, sustained efforts which includes bridging evidence and action and ensuring equitable access and improved health and wellbeing.

Scientific and professional societies can play a role in supporting and driving advocacy. The International Society for Pharmacoepidemiology (ISPE) provides a global forum for scientific exchange, collaboration, and methodological development and for the development of policy, education, and advocacy for the field of pharmacoepidemiology [6]. Through its strategic activities [7], the Society contributes to discussions on the use of pharmacoepidemiologic methods and real‐world evidence (RWE) generation for public health, regulatory science, and health policy decision‐making [8].

In this paper, we outline how the ISPE advances public health advocacy through the application of pharmacoepidemiology by identifying priority issues, developing methodological standards, contributing to public consultation processes, building capacity, and fostering strategic collaborations that support the translation of evidence into policy and practice. By highlighting these efforts, the manuscript serves as both a descriptive summary and a call to action by raising awareness of the Society's advocacy opportunities and encouraging active, sustained member's participation. Such engagement is essential, as ISPE's advocacy framework is fundamentally member‐driven. Beyond the ISPE audience, and by providing concrete examples of organizational structures, processes, and advocacy activities, the authors also seek to inspire and inform advocacy efforts within other international scientific and professional societies. This manuscript was endorsed by ISPE.

## 2 How Is Public Health Advocacy Through Pharmacoepidemiology Accomplished by ISPE?

ISPE's activities related to advocacy are structured around processes intended to support the generation of pharmacoepidemiologic evidence for public health‐relevant outputs. These activities span a continuum that includes from early horizon scanning (a structured activity whose objective is to detect and analyze at an early stage emerging “game changers” that could have significant impact on society and policy) [9] and the identification of emerging issues, participation in public consultations of policies in various jurisdictions, and collaboration with external organizations such as the European Network of Centres for Pharmacoepidemiology and Pharmacovigilance (ENCePP), the Council for International Organizations of Medical Sciences (CIOMS), and the International Network for Epidemiology in Policy (INEP), among others.

**FIGURE 1:**
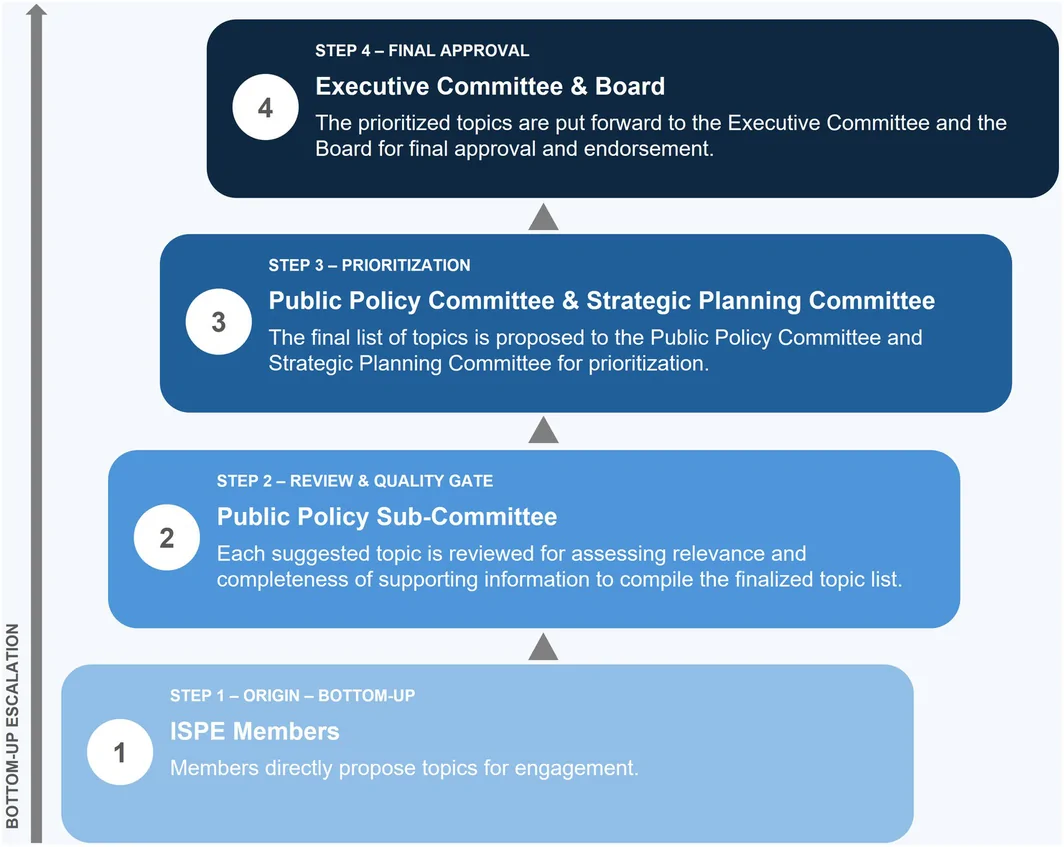
ISPE's member‐driven path to advocacy.

A key feature of this approach is the Society's governance structure, in which topics for engagement are proposed by members directly (bottom‐up approach). All suggested topics undergo a thorough review by the Public Policy Sub‐committee for their relevance and completeness of supporting information, and a final list of priority topics is proposed to Public Policy Committee (PPC) and Strategic Planning Committee (SPC) for endorsement before putting these topics forward to the Executive Committee and the Board. This process is designed to ensure that activities reflect a range of members' perspectives and are aligned with the Society's mission and available resources (Figure 1).

ISPE engages in advocacy through several types of activity, illustrated below:

### 2.1 Identification of Long‐Standing and Emerging Issues for Advocacy

The Society conducts structured horizon scanning to identify issues of potential relevance to pharmacoepidemiology and public health.

It occurs in structured and systematic processes through two mechanisms:

1. A biannual evaluation and identification of emerging topics and issues for advocacy [10]: ISPE's PPC conducts biannual horizon scanning and summarizes proposed topics and issues as submitted by ISPE members for prioritized discussions at ISPE's SPC. An *emerging issue* (https://www.pharmacoepi.org/strategic‐initiatives/ispe‐emerging‐advocacy‐issue‐checklists/) is defined as a topic of relevance to pharmacoepidemiology that is new to ISPE or which requires significant further development and guidance in terms of definition or methodological practices for appropriate application. Issues for advocacy are defined as those which require ISPE's consideration for advocacy aligned with its mission and goals and are considered actionable or otherwise can be influenced by ISPE advocacy efforts [10].
2. An ongoing opportunity for any member of ISPE to propose a potential strategic effort throughout the year [8]. Long‐term strategic efforts are reviewed by the SPC with a recommendation provided to the Executive Committee whereas short‐term strategic efforts are directly reviewed by the Executive Committee.

Issues for advocacy can be long‐standing or emerging. In both processes, the final decision on prioritization and selection of topics for actionable planning as part of ISPE's goals is made by the Executive Committee and Board based on considerations of public health importance, potential for impact, and anticipated resources needed. As part of this process, topics are assigned to one of three priority levels: (1) new strategic efforts supported and monitored by the Board; (2) non strategic activities that may be supported by individual ISPE Communities (e.g., regional or Special Interest Groups [SIGs]); or (3) topics for which ISPE engagement is not recommended.

For example, an emerging challenge identified by the ISPE Databases SIG was how to increase consistent reporting on data diversity which is required to enhance study interpretability and support replicability. In response, the *DIVERSE* framework [11], based on a systematic scoping review, was developed and has been operationalized into a tool now used for that purpose. Likewise, the Reporting Recommendations Intended for Pharmaceutical Risk Minimization Evaluation Studies: Standards for Reporting of Implementation Studies Extension (RIMES‐SE) was developed within ISPE. The RIMES‐SE takes an implementation science approach and considers the clinical context of medicines use as crucial to understand whether and why risk minimization interventions are effective or do not deliver the intended outcomes. It aims to ensure that all study information relevant to implementation and clinical context is included in the study report [12] and is among the checklists now encouraged for use by EU regulatory guidance to strengthen the quality, evidence and interpretability of study results for decisions on risk minimization interventions [13]. Similarly, an emerging issue identified by the ISPE Digital Technology and Artificial Intelligence (AI) SIG concerns the ethical implications of applying AI to real‐world data in the generation and interpretation of pharmacoepidemiologic evidence. In response, the SIG proposed a webinar and debate on ethics in AI‐supported pharmacoepidemiology spanning data, study conduct, and publication, addressing transparency, dataset representativeness and hidden bias, explainability and reporting expectations, and governance and human oversight in AI‐supported evidence generation.

### 2.2 Scientific and Methodological Contributions

Developing and shaping scientific standards by driving methodological innovation and capacity—strengthening remains one of ISPE's core functions. As the field of pharmacoepidemiology, real‐world data (RWD) and real world evidence (RWE) evolve, ISPE plays a critical role in identifying methodological gaps that may affect the quality, interpretability, and credibility of evidence used to inform public health and regulatory decision‐making.

For example, ISPE members have made major scientific and methodological contributions by developing and advancing widely used standards that underpin pharmacoepidemiology and RWE research. These include foundational guidance such as the Guidelines for Good Pharmacoepidemiology Practice (GPP), as well as more recent frameworks like the HARmonized Protocol Template to Enhance Reproducibility (HARPER), participation in collaborative initiatives such as Structured Template and Reporting Tool for Real‐World Evidence (STaRT‐RWE) [14], which promote transparency, reproducibility, and rigor in study design, conduct, and reporting, a reporting guideline specifically designed for pharmacoepidemiologic research that uses routinely collected health data, such as electronic health records, administrative claims, and registries (RECORD‐PE) [15] and frameworks for combining evidence from randomized and nonrandomized studies for decision‐making [16]. Collectively, these efforts have strengthened the credibility of pharmacoepidemiology research and supported its use in regulatory, policy, and clinical decision‐making. In addition, ISPE webinars addressing public health threats such as climate change convene experts from pharmacoepidemiology, environmental epidemiology, pharmacovigilance, and pharmaceutical engineering to define key challenges and identify strategies for action.

### 2.3 Public Commentary

ISPE contributes to public scientific and policy consultations in various jurisdictions by providing collective input on regulatory and scientific policy documents, particularly in circumstances of urgency [17] or broad societal impact. ISPE's official response is led by convening ISPE working groups with expertise in a related field to lead the development of public statements on behalf of ISPE. The endorsement process of these statements is coordinated by the PPC through a stepwise process of full membership review, and formal endorsement via the Board (Table 1 provides examples of ISPE 2025 activity).

**TABLE 1:** Summary examples of ISPE public commentary to US draft regulatory guidance in 2025 public commentary to draft regulatory guidance in 2025.

1. Considerations for the use of artificial intelligence (AI) to support regulatory decision‐making for drug and biological products [18]
The Society's feedback emphasized several key recommendations to strengthen the draft guidance including aligning AI/machine‐learning terminology and encouraging practical application of the framework in real‐world settings, especially in pharmacovigilance and drug safety. To ground the risk‐based framework in practical application, we proposed real‐world use cases—such as AI‐driven pharmacovigilance systems that analyze electronic health records to detect adverse drug reactions—highlighting how the framework could be applied in post‐market safety surveillance. We also stressed the importance of lifecycle management, especially for self‐evolving systems, and recommended the establishment of a centralized, public‐facing repository of validated AI models, like the EU AI Act's registry for high‐risk models, to enhance transparency and reusability. Lastly, we called for greater attention to model provenance and governance, encouraging the FDA to consider frameworks from organizations like the National Telecommunications and Information Administration and to set clear expectations around documentation, versioning, and traceability that are proportional to the model's risk and regulatory impact.
2. Considerations for the study of sex differences in the clinical evaluation of medicinal products [19]
Several key points were raised in ISPE's response, of which the distinction between sex and gender, the matter of lack of representation of women in clinical trials, and the fact that limited or lack of data on sex differences should not be translated as the absence of sex differences. Further, emphasis was placed on a historic inattention paid to female data in specific diseases (e.g., cardiovascular and Parkinson's disease) due to a lack of understanding of biological differences and the importance of applying specific statistical concepts to fully support hypothesis‐driven analyses powered to conduct sex‐based analysis in clinical trials. In addition, ISPE highlighted issues, such as the underrepresentation of women in the training, testing, and validation of AI models, and the types of biases—whether intentional or unintentional—that can lead to inaccuracies and further threaten to address the needs of marginalized trial participants and to adversely affect downstream users of clinical data.
3. Considerations for the collection and submission of patient preference information (PPI) across the total product lifecycle [20]
Comments from ISPE members included requests for additional clarity regarding the Agency's recommendations to ensure that sponsors' investment in this research is valuable for decision‐making. ISPE's comments focused on areas in which sponsors may seek clarification including: Context beyond benefit risk assessment in which PPI can be useful to support sponsors' evidence planning Guidance or examples to guide sponsors in the design and execution of formative qualitative preference research Context and applications along with the total product lifecycle in which preferences of cohorts other than (or in addition to) patients would be considered by FDA Additional methodological guidance, including for example, the types of preference heterogeneity analysis and the selection and interpretation of assessments of response consistency

A recent example illustrates how this process contributes to evidence‐to‐policy translation over time. In February 2024, drawing on its Medical Device SIG and its broader membership expertise in RWD methods, ISPE submitted comments on the FDA draft guidance Use of Real‐World Evidence to Support Regulatory Decision‐Making for Medical Devices [21]. Among other points, ISPE recommended broadening the description of disease‐specific data sources beyond rare or poorly understood conditions, clarifying the level of participant‐level data access expected of sponsors when working with aggregated or federated data, and strengthening recommendations on device identification. The final guidance, issued in December 2025, incorporated revisions consistent with several of these recommendations [22]. The description of disease‐specific RWD sources was broadened to apply to diseases generally rather than only rare or poorly understood conditions. The reliability section was revised to clarify that a sponsor's inability to obtain participant‐level data does not preclude FDA evaluation, provided the sponsor describes how this limitation affects the resulting evidence. Because final guidance reflects input from many contributors, individual changes cannot be attributed to a single source. However, the alignment between ISPE's recommendations and the revised guidance nonetheless illustrates how structured public commentary participates in shaping regulatory standards for RWE.

## 3 Strategic Partnerships and Other Collaborations

The Society strategically engages with external organizations to contribute to joint initiatives, including publications and methodological efforts aimed at strengthening research practices and supporting evidence use in policy contexts. These collaborations reflect a broader effort across the field to improve transparency, reproducibility, and the application of RWE.

To support this goal, the ISPE Board selects members with leadership experience to act as strategic liaisons with external organizations to identify opportunities for collaboration, including advocacy efforts.

Examples of these collaborations are position statements developed in collaboration with the INEP [23] emphasizing the importance of conflict‐of‐interest disclosure in epidemiological research to safeguard scientific integrity, as well as leadership of an INEP article establishing the concept of epidemiological literacy and calling for its strengthening across policy and public health communities [24].

Other collaborations include joint advocacy to advance understanding of pharmacoepidemiology and other outcomes research, such as the ongoing RWE Transparency Initiative involving ISPE, the Professional Society for Health Economics and Outcomes Research (ISPOR), the Duke‐Margolis Center for Health Policy, and the National Pharmaceutical Council which has led to the development of a RWE registry for studies before their start to increase reproducibility [25]. Another effort is the development of the HARPER template which was recognized in the International Council for Harmonization (ICH) guideline [26]. This ICH‐M14 guideline is now recommended by regulators [27] and payers [28].

## 4 ISPE's Long‐Term Vision and Strategic Planning

Overall, these activities illustrate examples of how a scientific professional society may structure efforts to support the interface between evidence generation and its use in policy and practice. In this framing, advocacy is understood as a set of actions embedded within scientific collaboration, methodological development, and stakeholder engagement.

Advancing advocacy in pharmacoepidemiology over the long‐term is central to ISPE's mission to improve public health through scientific exchange, methodological innovation, education, leadership, and policy engagement. The ISPE Strategic Plan (2024–2029) explicitly positions advocacy as a core mechanism for supporting and translating methods and pharmacoepidemiology into public health policy and clinical practice across diverse global contexts [29]. To operationalize this vision, ISPE is committed to completing at least one advocacy‐aligned activity annually in at the minimum of 90% of its ongoing external partnerships, embedding advocacy within sustained scientific collaboration with external organizations and ensuring that evidence generation is closely linked to real‐world decision‐making needs, serving public health for all.

Importantly, ISPE is positioning itself to expand and sustain its advocacy activities beyond the 2025–2029 strategic plan horizon, reflecting a long‐term commitment to strengthening the role of pharmacoepidemiology in public health and policy.

This long‐term vision is also tightly linked to capacity building and members' empowerment. By expanding opportunities for engagement, leadership development, and cross‐sector collaboration—particularly for early‐ and mid‐career professionals and members from underrepresented regions—ISPE seeks to cultivate a diverse, globally distributed community capable of shaping and leading evidence‐informed policy discussions. Through mentorship, training initiatives, and inclusive governance, the Society aims to ensure that the next generation of pharmacoepidemiologists is equipped not only with technical expertise but also with the skills needed to engage effectively in public advocacy.

Ultimately, ISPE envisions advocacy as a sustained, reciprocal process that both informs and is informed by evolving public health needs. By embedding advocacy within its scientific mission and fostering a culture of engagement, the Society aims to enhance the relevance, credibility, and impact of pharmacoepidemiology—ensuring that it remains a critical driver of safe, equitable, and evidence‐based use of medicines and medical devices worldwide.

## Funding

The authors have nothing to report.

## Disclosure

The views in this article are the authors' personal views and must not be taken as the views of their respective employers.

## Conflicts of Interest

G.S. is employed and holds Cytel shares and nonpaid positions at ISPE (co‐chair of PPC, Board member) and to a European‐funded AI trial in breast cancer (https://cinderellaproject.eu/). J.D.‐D. is Founder and Principal of Black Swan Causal Labs. He was an employee at ModernaTX Inc. during the early development of this manuscript. M.E.R. is an employee of Merck Sharp & Dohme LLC, a subsidiary of Merck & Co. Inc., Rahway, NJ, USA, owns stock in Merck & Co. Inc., Rahway, NJ, USA and the chair of PPC when the manuscript was developed. M.S.G. is a former employee of Bayer AG and GSK and holds shares in GSK. R.G. is employed by ARS Toscana, a publicly owned research center that conducts studies funded by private and public institutions and compliant with the ENCePP Code of Conduct. M.E.R. and M.S.G. are editorial board members of Pharmacoepidemiology and Drug Safety, however they were excluded from editorial decision‐making related to the acceptance of this article for publication in the journal. M.E.R. is a past ISPE President (2024–2025) and current member of the Executive Committee. The other authors declare no conflicts of interest.

## Data Availability

Data sharing is not applicable to this article as no datasets were generated or analyzed during the current study.
